# Dynamical footprint of cross-reactivity in a human autoimmune T-cell receptor

**DOI:** 10.1038/srep42496

**Published:** 2017-02-14

**Authors:** Amit Kumar, Francesco Delogu

**Affiliations:** 1 Department of Mechanical, Chemical and Materials Engineering, University of Cagliari, via Marengo 2, 09123 Cagliari, Italy; 2 Biosciences Sector, Center for advanced study research and development in Sardinia (CRS4), Loc. Piscina Manna, 09010 Pula, Italy

## Abstract

The present work focuses on the dynamical aspects of cross-reactivity between myelin based protein (MBP) self-peptide and two microbial peptides (UL15, PMM) for Hy.1B11 T-cell receptor (TCR). This same TCR was isolated from a patient suffering from multiple sclerosis (MS). The study aims at highlighting the chemical interactions underlying recognition mechanisms between TCR and the peptides presented by Major Histocompatibility Complex (MHC) proteins, which form a crucial component in adaptive immune response against foreign antigens. Since the ability of a TCR to recognize different peptide antigens presented by MHC depends on its cross-reactivity, we used molecular dynamics methods to obtain atomistic detail on TCR-peptide-MHC complexes. Our results show how the dynamical basis of Hy.1B11 TCR’s cross-reactivity is rooted in a similar bridging interaction pattern across the TCR-peptide-MHC interface. Our simulations confirm the importance of TCR CDR3α E98 residue interaction with MHC and a predominant role of P6 peptide residue in MHC binding affinity. Altogether, our study provides energetic and dynamical insights into factors governing peptide recognition by the cross-reactive Hy.1B11 TCR, found in MS patient.

Multiple sclerosis (MS) is a chronic inflammatory and degenerative disease of the central nervous system^1^, affecting more than 2.5 million people worldwide^2^. MS exhibits a heterogeneous geographical pattern affecting populations across the globe. In particular, it is more common far from the equator and shows latitude gradient^3^.

MS involves an abnormal response of the human body’s immune system directed against brain and spinal cord. In particular, the immune system attacks myelin, i.e. the protective substance covering and insulating nerve fibers. The disease owes its name exactly to the sclerosis formed by damaged myelin^4^. Damage, or destruction, of any part of the myelin sheath or nerve fibers cause the distortion, or interruption, of the nerve impulses that travel to and from brain and spinal cord. Eventually, nerve fibers themselves can deteriorate or suffer from permanent damage. A wide variety of symptoms determined by the location of lesions within the central nervous system can arise^5^, ranging from loss of sensitivity and changes in sensation to pain, muscle weakness, difficulty in moving and sight and speech problems^6^.

Overall, the exact cause of MS disorder still remains elusive^7^, with the disease linked to both genetic^8^,^9^ and environmental^10^,^11^ factors. Experimental and clinical studies have provided evidence reinforcing the hypothesis that immune mechanisms are involved in the pathogenesis of inflammatory demyelination in MS^7^,^12^. In fact, the exact antigen that immune system cells are sensitized to attack has not been recognized yet, and this leads many experts to consider MS as an “immune-mediated” process, rather than an “autoimmune” disease. Nevertheless, multiple findings identified the human leukocyte antigen (HLA) class II system as the main genetic determinant region related to MS^8^,^13^,^14^. An important role of autoreactive T lymphocytes in the initiation and perpetuation of disease has also been suggested^2^,^15^.

T-cells form a subset of lymphocytes, critical for providing an adaptive immune response against invading pathogens^16^. In particular, the T-cell receptor (TCR) at the surface of T lymphocytes is a complex of integral membrane proteins that participates in the activation of T-cells in response to an antigen^17^. Stimulation of TCR is triggered after recognition of antigenic peptides presented by the major histocompatibility complex (MHC), corresponding to HLA in humans, located on the surface of antigen-presenting cells^18^. A successful TCR engagement initiates positive and negative cascades leading to T-cell activation, differentiation, proliferation and, finally, to a specific immune response to the invading pathogen^19^,^20^.

Peripheral T-cells are commonly trained to recognize a widest set of pathogen-derived epitopes while ignoring self-antigens^18^. However, there are also cases in which some TCRs escape this selection and are able to recognize self-antigens, thus initiating an autoimmune response and becoming self-reactive^21^. The term TCR cross-reactivity is associated exactly to the recognition of many different peptide antigens presented by the HLA of an individual^22^,^23^,^24^. The three-complementarity determining region (CDR) loops of the α and β chains present in TCR facilitate the recognition of peptide-HLA-II complex (Fig. 1)^25^. Majority of contacts with the bound peptide involve CDR3 rather than CDR1 and CDR2^26^.

Many experimental studies have shown the importance of TCR cross-reactivity in initiating adaptive immune response^27^,^28^,^29^. However, a direct correlation between TCR binding affinity^30^,^31^,^32^ and potency of T-cell activation has not been proved, and the overall process is still poorly understood^33^. Multiple mechanisms of T-cell receptor cross-reactivity have been proposed on the basis of the solved three-dimensional structures for the tri-molecular complex TCR-peptide-HLA^34^. Specifically, induced fit^35^, differential TCR docking^22^, structural degeneracy^36^, molecular mimicry^27^,^37^, and antigen-dependent tuning of peptide-HLA flexibility^28^, were proposed. Around twenty three-dimensional structures for the TCR-peptide-MHC-II complex structures have been determined providing structural insights into MHC restriction^18^. In a previous study, investigators isolated an autoimmune Hy.1.B11 TCR from a MS patient^38^. This TCR was initially found to be specific for myelin basic protein (MBP) peptide bound to the HLA-DQ1 (DQA1*0102-DQB1*0502) class II protein. However, it has been observed that the same Human Hy.1.B11 T-cell clone is not only activated by MBP, but also by other distinct microbial peptides^39^. The structural basis of cross-reactivity displayed by human Hy.1.B11 TCR between MBP and microbial peptides from UL15 terminase protein of *Herpes simplex virus* and phosphomannomustase protein of *Pseudomonas aeruginosa* has been recently investigated^25^. The crystallographic structures of these complexes revealed a common tilted TCR binding topology onto the peptide-MHC surface, pointing out a dominant involvement of CDR3*α* residues towards both self and microbial peptide. This aspect clarified the docking geometry and static interaction picture between TCR and peptide-MHC molecule, providing strong indication about the role of local molecular configurations in cross-reactivity. However, an exclusively static conceptual framework can be hardly thought to allow a satisfactory understanding of the complex mechanisms underlying cross-reactivity. Accurate dynamical information is needed as well to unveil the role of atomic and molecular motion in the involvement of CDR residues.

Computational molecular dynamics (MD) simulations can provide the necessary finite temperature atomistic level description dynamic changes of TCR in the presence of peptide-MHC complex, which could be useful to understand better engagement of TCR by the immune system. MD simulations have been already employed to investigate, for instance, the dynamics of tri-molecular TCR-peptide-MHC-I complexes, with either altered/different peptides^40^,^41^,^42^, or different MHC’s^43^ or different TCR’s^44^. Yet, until date only one computational study has been performed on the TCR-peptide-MHC-II complex^45^, wherein the authors investigated the energetic and flexibility properties of the complex with a native peptide as well as for twelve mutations introduced in the peptide.

In the present study we perform and analyze MD simulations of the three tri-molecular complexes relevant to observed TCR cross-reactivity in a MS patient. To further probe the role of peptide-MHC complex dynamics in TCR cross-reactivity, we performed additional simulation of three peptide-MHC complexes in the absence of TCR structure.

Our simulations highlight the important energetic role of CDR3*α* TCR loop in binding to HLA-DQ1-peptide complexes; in particular the key contribution to peptide recognition by CDR3*α* E98, a residue that is conserved in the three tri-molecular complexes. Furthermore, we found a new interaction between another TCR loop CDR2β D55 residue and HLA-DQ1α K39 residue, located outside the peptide-binding groove, to constitute a conserved anchor point for docking TCR on to MHC class II protein. Altogether, these results explain the dynamical basis for cross-reactivity between the MBP self-peptide and the two microbial peptides for Hy.1B11 TCR.

## Results

For the three peptides (MBP, UL15, PMM), we performed TCR-pMHC MD simulations and pMHC MD simulations, each 110 ns in length. The stability of complexes was checked by evaluating the root mean square deviation of the C-alpha atoms (Supplementary Materials). The convergence of MD simulations (Supplementary Materials) was estimated using a novel Good-Turing statistical approach^46^.

### Molecular interactions of TCR with HLA-DQ1 bound peptides

**Figure 1:**
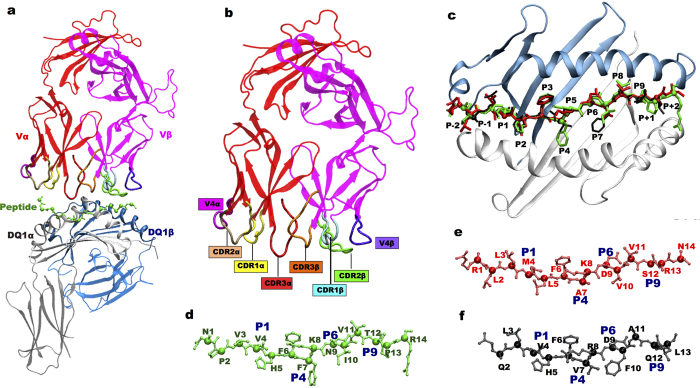
Peptide cross-reactivity of Hy.1B11 TCR. (**a**) Structure of TCR-MBP-HLA-DQ1 complex: in red TCR Vα; in pink TCR Vβ; MBP peptide in green; HLA-DQ1α in grey; HLA-DQ1β in light blue. (**b**) Complementary determining regions (CDR’s) of TCR. (**c**) HLA-DQ1 peptide binding groove with the three peptides and their pockets. (**d,e,f**) Peptides structures with residues named using single letter nomenclature: MBP in green; PMM in red and UL15 in black, and the pockets P1, P4, P6 and P9 are indicated in blue.

**Figure 2:**
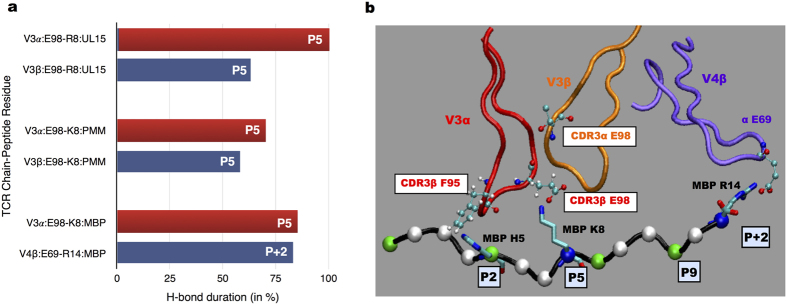
Hydrogen bond interaction TCR and peptide residues. (**a**) H-bond duration between TCR-peptide residues in %. (**b**) Interaction picture between TCR and MBP peptide.

The persistent H-bond interaction evaluated between the HLA-DQ1 bound peptide residues and TCR residues survived for than 60% of the MD simulations. H-bond interactions of TCR with MBP and the two microbial peptides (UL15, PMM) were characterized by a unique pattern of pair-wise contact between CDR3 Vα E98 and pocket 5 (P5) K8/R8 of the peptide complexes (Fig. 2). A marked difference in H-bond interaction pattern between the self and microbial peptide complexes was observed. In the two microbial peptide complexes, the same peptide residue K8/R8 (Fig. 1e,f) shared a common interaction pattern with CDR3β D97 residue. While, a specific pair-wise contact between fourth hyper variable loop V4β residue E69 and R14 of MBP peptide complex was observed (Fig. 2b).

Persistent stacking interactions considered between the TCR and peptide residues, survived for more than 40% of MD simulations. CDR3α F95 residue formed a persistent stacking interaction with the P3 (Phe6) PMM peptide residue and with the P2 (His5) MBP peptide residue, respectively. However, in the UL15 peptide complex no persistent stacking interaction involving CDR3α F95 was noted.

### Interactions of TCR with HLA-DQ1

**Figure 3:**
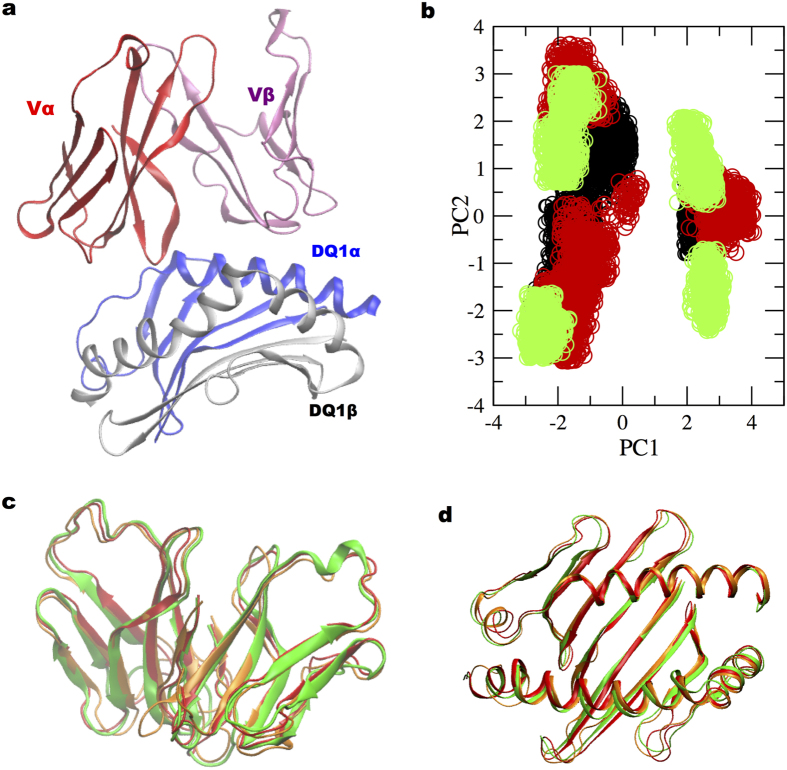
dPCA on the TCR-MHC class II for the three molecular complex systems. (**a**) TCR residues α-chain (24:104) in red and β-chain (24:103) in magenta; HLA-DQ1 α-chain (5:76) in blue and β-chain (7:90) in grey. (**b**) The two dimensional point maps correspond to projection of dihedral angles (phi, psi) fluctuations (from MD simulation trajectory) on the plane defined by first two principal components: (i) black: UL15, (ii) red: PMM and (iii) Green: MBP peptide complexes. Superposition of (**c**) TCR Vα and Vβ domains and (**d**) HLA-DQ1 α1 and β1 domains, for the three-peptide complexes.

The residues of TCR/HLA-DQ1 complex (Fig. 3a) were selected to perform dihedral angle principal component analysis^47^ (dPCA) for the three complexes on the 5500 snapshots extracted from MD simulation trajectory. The dihedral angles of TCR-MHC residues were projected onto the first two principal components (PC) for each trajectory snapshot from MD simulations for the three complexes (Fig. 3b). Each point in the plot (Fig. 3b) represents a specific configuration explored by the TCR-MHC complex during MD simulations. Projection of dihedral angle fluctuations along the first two principal components in three peptide complexes (UL15, PMM, MBP, Fig. 3b), suggested a more limited phase space exploration in the microbial peptide complexes with respect to MBP complex.

For further investigation, we performed cluster analysis (see Methods) and identified the most populated configurations in the three complexes and subsequently obtained their corresponding representative structures. The difference between the representative TCR-MHC configurations in the microbial peptide complexes to that in the MBP peptide complex case was evaluated by calculating the RMSD values (Table 1).

We found the TCR-MHC class II structures in microbial peptide complexes and MBP complex to overlap quite nicely (Fig. 3c,d) with an RMSD difference less than 2 Å. This observation is consistent with comparable buried surface area values in the three complexes (Table 1).

Persistent hydrogen bonded interactions between the TCR and HLA-DQ1 residues are reported in Table 2. We found both conserved and non-conserved interactions between the MBP peptide and two microbial peptide complexes. Overall, we found a higher number of interactions between the TCR residues and DQ1 α1 helix residues, while peptide specific TCR interactions with DQ1 β1 helix residue was noted only for the microbial peptide complexes.

**Figure 4:**
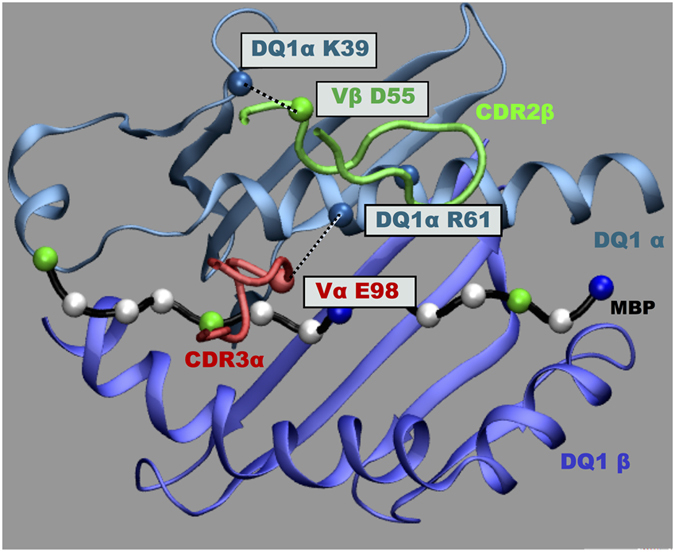
Conserved TCR-MHC contacts. H-bond interactions and placement of CDR2β loop over DQ1 α-helix. The participating residues are boxed, and H-bond is denoted by dashed line.

Remarkably, the titled orientation of TCR limits its interactions with DQ1 β1 helix residues. Moreover, CDR2β loop overlays the central portion of DQ1 α1 helix and Vβ D55 residue involved in H-bond interaction with DQ1 α1 residue K39 located outside the peptide-binding groove (Table 2, Fig. 4).

### HLA-DQ1 interactions with bound peptides in the TCR complex

**Figure 5:**
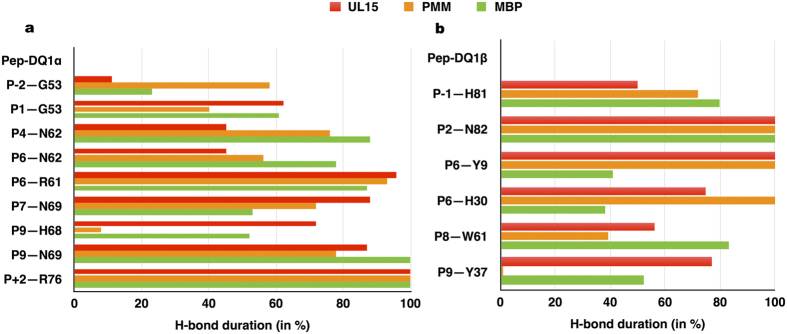
Hydrogen bonded interactions between peptide and HLA-DQ1 residues. In (**a**) peptide-DQ1α interacting pairs, and in (**b**) peptide-DQ1β interacting pairs.

We analysed MD simulation trajectory for the three TCR-pMHC complexes and report only the persistent H-bond interactions observed between the peptide-MHC pairs (Fig. 5). In general, we found DQ1 α-chain residues (Fig. 5a) to display a predominant involvement in H-bond interactions with the peptide residues. This observation derives higher number of interacting pairs with respect to that of β-chain (Fig. 5b). Even tough the two microbial peptides are quite different in their sequence from MBP peptide, the majority of the H-bond interactions between MHC residues and specific positioned peptide residues was conserved.

Subsequently, we also analysed persistent stacking^48^ interactions between the peptide residues and the two chains of MHC protein. A conserved pair-wise stacking contact between DQ1 α-chain residue R61 and pocket 3 of the peptide cases was observed. On the other hand, we also found peptide-specific stacking interaction pattern involving DQ1 β-chain residues.

### Entropy and interaction energy estimation

**Figure 6:**
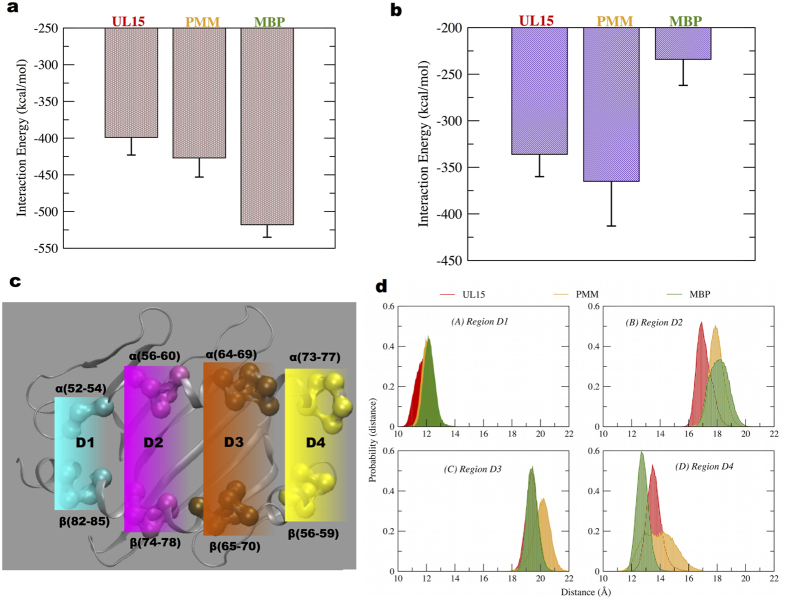
Interaction energy plots and HLA-DQ1 groove width analysis. (**a,b**) Interaction energy corresponds to non-bonded energy values comprising of Van der Waals and electrostatic energy between: (**a**) TCR and pep-MHC and in (**b**) peptide and MHC. (**c**) Dissection into four regions D1 to D4, (d) center of mass distance variation in the four different regions, MBP complex in green, UL15 complex in red and PMM complex in orange.

Configurational entropy^49^ values calculated for HLA-DQ1 peptide binding groove residues and for the TCR-MHC components respectively, showed highest value for the microbial peptide PMM complex, while the self-peptide MBP complex displayed an intermediate value. The calculated interaction energy of TCR with peptide-DQ1 complexes showed highest value for DQ1/MBP than DQ1/PMM and DQ1/UL5 cases (Fig. 6a). A similar trend was also noted by better binding affinity (higher interaction energy value) of MBP peptide for TCR (−206 kcal/mol), compared with the two microbial peptide cases (−125 kcal/mol). However, observing binding affinity of peptides for DQ1 alone, we found the lowest interaction energy value for MBP complex (Fig. 6b).

### HLA-DQ1 peptide binding groove width fluctuation in TCR simulations

To probe the importance of binding groove flexibility at a local level description, we dissected the groove into four regions^50^ (D1–D4, Fig. 6c). The center of mass distance variation between the DQ1 α-chain and β-chain residues was selected as a parameter to monitor the distance fluctuations in these four regions.

In region D1, the distance profile distribution is quite similar for the MBP and PMM peptide bound cases, while it is slightly left shifted in the UL15 peptide bound case (Fig. 6d). In region D2, we note a broader distribution for the MBP case; while a narrow distribution in the microbial peptide cases and left shifted peak distribution was noted in the UL15 peptide case. In region D3, we note a perfect overlap this time between MBP and UL15 peptide cases, while a broader and right shifted distribution in the PMM peptide case. In region D4, we note a left shifted distance peak distribution for the MBP case, with respect to the two microbial peptide cases. Moreover, we observed region D4 to be very flexible for the PMM bound case, with fluctuation in the range 12–16 Å. Overall, we found MHC binding groove displayed higher flexibility in PMM bound case, in particular in the regions D3 and D4. This finding is consistent with a higher value of configurational entropy observed (Table 3) for the MHC binding groove in the PMM bound case.

### Comparative study for peptide-MHC complex simulations without TCR

**Figure 7:**
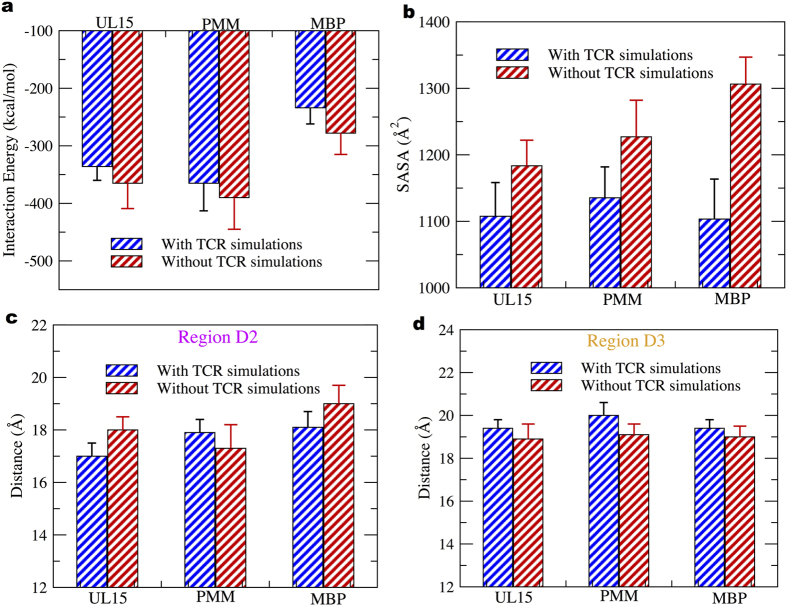
HLA-DQ1 binding groove analysis in the presence and absence of TCR. In (**a**) peptide-HLA-DQ1 interaction energy corresponds to non-bonded energy values comprising of Van der Waals and electrostatic energy (**b**) HLA-DQ1 peptide binding groove SASA. In (**c,d**) average distance values in region D2 and D3 of the HLA-DQ1 binding groove.

To address the role of peptide/HLA-DQ1 complex dynamics in T-cell receptor cross-reactivity we performed additional simulations without TCR and compared the results with simulations performed with the TCR. The average interaction energy values calculated from MD simulations suggested a better peptide-MHC binding in simulations performed without the TCR (Fig. 7a).

Furthermore, the average solvent accessible surface area (SASA)^51^ values of HLA-DQ1 binding groove showed an increased value in all three peptide cases for TCR free simulation cases (Fig. 7b). The maximum increase in average value of SASA (~15%) was noted in MBP peptide complex. Binding groove width analysis on a local scale was performed in the absence and presence of TCR on the MD simulations trajectories. We found significant variation in average distance values in the regions D2 (Fig. 7c) and D3 (Fig. 7d) in all the three-peptide complexes. In region D2 (Fig. 7c) we found that groove width increases when not bound to TCR, in the UL15 and MBP peptide complexes. However, for all the peptide cases, in region D3 we note the groove to be slightly narrow in the absence of TCR (Fig. 7d). Only in the MBP peptide case, we observed an opening (~1.5 Å) of HLA-DQ1 region D4 when not bound to TCR (Supplementary Materials).

To understand better the differences noted in the binding groove dynamics between the TCR bound and unbound cases, we examined the peptide-MHC interaction network (Table 4). In all the peptide complex cases, we note a striking absence of H-bond interaction between DQ1 αR61 and P6 peptide residue in the simulations performed without TCR. On the other hand, we observed a novel H-bond interaction for the unbound TCR simulations between DQ1 βE66 and P5 peptide residue, in all the peptide complexes. Overall, we found an increase (~6–8%) in the HLA-DQ1 binding groove entropy values in the TCR unbound cases, suggesting a much more flexible binding groove in the absence of TCR.

## Discussion and Conclusions

The objective of our study has been to provide dynamical insight into Hy.1B11 TCR cross-reactivity between MBP self-peptide and two microbial peptides while bound to HLA-DQ1 complex. To address this issue, we performed molecular dynamics simulations on available experimental atomistic model of Hy.1B11 TCR from a patient with relapsing-remitting MS disease. The central role of TCRs is to recognize the peptide presented by MHC molecules and provide an immune protection against foreign peptides^52^.

It is interesting to note that the total number of possible peptides of 14-mer length that can be constructed from the 20 amino acids are of order ~10^18^. Without entering in the details of the peptide and complex structural constraints, even assuming only a very low percentage of this peptide repertoire to bind to MHC class II molecules, the possible number of peptides is still many order of magnitude greater than the theoretical number of possible TCRs in humans (~10^8^). Thus, the bandwidth of TCR cross-reactivity is inevitable to compensate this disparity and to provide an immune cover for vast number of peptide-MHC complexes^53^. TCR cross-reactivity can have both positive and negative implications. On one hand, a positive implication can be providing polyclonal recognition of peptide-MHC molecules, thus providing immune cover against pathogens that escapes recognition. While, on other hand, a negative consequence can be for causing autoimmune diseases^53^.

Previous crystallographic studies of two human TCR’s^36^,^54^ from MS patients, displayed between them a different binding geometry to the peptide-MHC complex, but a common CDR footprint displaced towards the N-terminal of the bound MBP peptide. In our study, the Hy.1B11 TCR structure not only displays a different binding geometry with respect to these human TCR structures, but also a different CDR footprint. In particular, with CDR2β loop to overlay onto the central portion of HLA-DQ1 α-helix (Fig. 4) and CDR3α, CDR3β chains positioned towards the center portion of peptide binding groove.

Our MD simulations confirmed the energetic role of CDR3α E98 residue^38^ in interaction with the P5 (Lys/Arg) peptide segment (Fig. 2). Mutation of CDR3α residue E98 to alanine (A98) resulted in a significant reduction in interaction energy value (Supplementary Material) between TCR and MBP-MHC residues with respect to wild-type case. These observations are consistent with previous experimental data, wherein alanine substitution of P5 resulted in a complete loss of activity and CDR3α E98A mutation was particularly severe to HLA-DQ1 peptide binding^25^. Previous structural investigation of Hy.1B11 TCR for HLA-DQ1-peptide (MBP, UL15, PMM) complexes^25^ suggested a dominant role of CDR3α F95 residue for recognition of both self-peptide (MBP) and microbial peptides (UL15, PMM). No such predominant role is observed from our MD simulations. However, we do observe persistent stacking interactions between CDR3α F95 and with peptide pockets P2 and P3 in the MBP and PMM peptide cases, respectively.

Recent experimental study^29^ investigated the TCR-peptide-MHC cross-reactivity for nine peptides with limited sequence similarity and noted a consistent TCR-MHC interaction mode in all peptide complexes. With a similar strategy, we investigated how the TCR-MHC docking configurations changed during MD simulations by dPCA analysis and also evaluated the interaction mode for the three cross-reactive peptide complexes. Notably, the three different peptide complexes superimposed neatly (Fig. 3c,d, within a RMSD difference of 2.0 Å) and displayed similar values of total buried surface area, obtained from MD simulations (Table 1). Specifically, about MHC class II system one must consider that the peptide-binding groove is quite well characterized in literature and one can expect only local rearrangements in bound peptide conformations. Therefore, no drastic difference in the global conformation between the three peptide complexes is expected with respect to the starting crystal structures.

Moreover, a common interaction picture (Fig. 4) was noted in the peptide complexes, mediated through TCR Vα domain, in which CDR3α E98 formed H-bonds to R61 of HLA-DQ1α and through Vβ domain, in which CDR2β D55 formed H-bonds to K39 of HLA-DQ1α (Table 2). Indeed, surface plasmon resonance (SPR) experiments showed CDR3α E98-DQ1α R61 interaction to be energetically the most important interaction between the Hy.1B11 TCR and HLA-DQ1 residues^25^.

An energetic picture of cross-reactive Hy.1B11 TCR was obtained by performing interaction energy calculations to estimate the binding affinity of TCR for three DQ1-peptide complexes (Fig. 6a). MD simulations provided best interaction energy value of Hy.1B11 TCR for DQ1/MBP complex, than DQ1/PMM and DQ1/UL15 complexes. This trend in TCR binding affinity for DQ1-peptide complexes is in perfect agreement with SPR experiments^25^, which showed Hy.1B11 TCR higher binding affinity for DQ1-MBP complex. Furthermore, the same experiments showed the lower TCR affinity for the DQ1-microbial peptide complexes to be partially compensated by higher value of binding affinity of PMM and UL15 for DQ1, compared with the MBP peptide. To address the same, we evaluated the interaction energy values of DQ1 for MBP, UL15 and PMM peptides (Fig. 6b). Our data correlated well with the experimental data. Thus, supporting the importance of an energetic balance between both TCR affinity for DQ1-peptide and DQ1 affinity for peptide in Hy.1B11 TCR cross-reactivity. With the unique exception of P5, all other key anchor (pockets) peptide residues, contributed to the DQ1-peptide binding affinity (Fig. 5, Table 3). In spite of different anchor residues between the microbial peptides and MBP self-peptide, we note conserved interaction with DQ1 residues. Interestingly, the conserved peptide residue (Phe) at P3 position formed persistent stacking interaction with DQ1 residue αR61 (Table 3). The predominant role of peptide residue at P6 position in H-bond interaction with both α-chain (Fig. 5a) and β-chain residues (Fig. 5b) of DQ1 binding groove was observed. The two microbial peptides shared a common peptide residue (aspartic acid) at P6 position, different from asparagine residue in MBP. This difference at P6 position was reflected by a lesser persistent interaction between asparagine residue in MBP and residues Y9 and H30 of DQ1 β-chain (Fig. 5b), consistent with lower binding DQ1 affinity of MBP. The local fluctuations of the binding grove in the four regions highlighted similarity in width fluctuation between self-peptide MBP and the microbial peptides: in region D1 involving UL15, while in region D2 with PMM (Fig. 6b). A wider and flexible regions D3 and D4 in the PMM complex resulted in an overall higher value of entropy (Table 3).

To understand the role of peptide-MHC dynamics in Hy.1B11 TCR cross-reactivity, we performed additional simulations of the peptide-MHC complexes without the TCR. In the simulations without TCR, we observed an overall increase (6–8%) in HLA-DQ1 binding groove flexibility (Table 4), and the trend in local MHC width fluctuations to be inverted between regions D2 and D3. A slight but a significant difference in peptide-DQ1 binding affinity (Fig. 7a) and an increase in solvent accessible surface area (Fig. 7b) was observed. Interestingly, in the simulations without TCR, we note absence of a conserved H-bond interaction between peptide residue at position P6 and residue R61 of DQ1 α-chain, and a new interaction between peptide residue at position P5 and residue E66 of DQ1 β-chain, in the three peptide-MHC complexes (Table 4). These observations confirm the role of TCR in bridging interaction between peptide position P6 and residue R61 of DQ1 α-chain. It is important to mention that in the TCR simulations, peptide residue at position P5 was the one involved in interactions with TCR residues. In summary, we found the presence of TCR to have an important impact on both local and global level description of peptide-MHC interactions.

**Figure 8:**
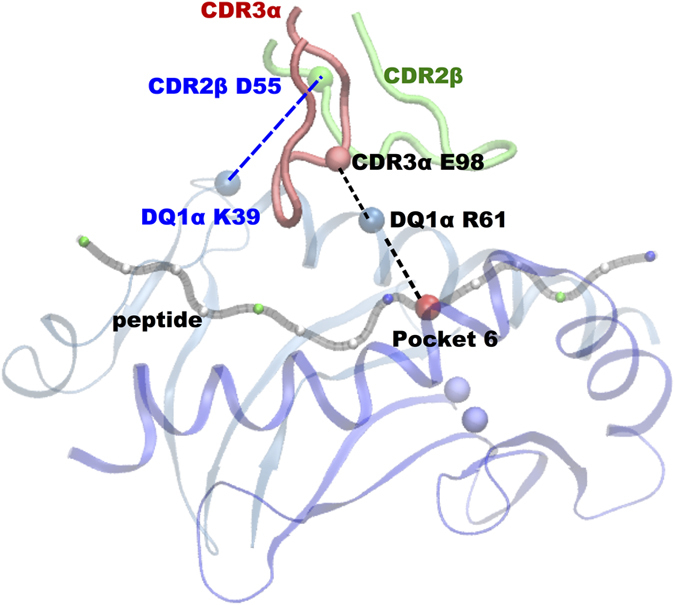
Bridging interaction across TCR-MHC-peptide interface determining Hy.1B11 TCR cross-reactivity. H-bond interactions are represented with dashed lines.

In conclusions, using MD simulations, we identified a bridging interaction involving CDR3α (E98) − DQ1α (R61) − peptide (P6) as an energetic hot spot on the TCR-peptide-MHC interface that contributes to Hy.1B11 TCR cross-reactivity (Fig. 8). We further identified a structurally relevant new H-bond interaction between CDR2β D55 and DQ1α K39 to constitute a key anchor point for interaction of TCR on to the MHC class II. Our findings confirm the energetic role of CDR3α residue E98 in Hy.1B11 TCR cross-reactivity.

Altogether, using MD simulations we were able to identify involvement of a small number of structurally and energetically important hot spots that provide dynamical insights into Hy.1B11 TCR cross-reactivity.

## Methods

### Model preparation

In the current study, we chose the available X-ray structures of the tri-molecular TCR-peptide-MHC-II complexes for the two microbial peptides UL15 (PDB id: 4MAY), PMM (PDB id: 4GRL) and one self-peptide MBP (PDB id: 3PL6) complex. The tri-molecular structures consisted of same Hy.1B11 TCR, same HLA-DQ1 protein complexed alternatively with the three peptides under investigation (Fig. 2). HLA-DQ1 protein is a heterodimer composed of two chains: α (DQA1*0102) and β (DQB1*0502); and the peptide-binding groove is formed from two non-covalently linked subunits of α1 (5–90 residues) and β1 (5–90 residues) chains. The missing hydrogen atoms in the X-ray structures of the three TCR-peptide-MHC-II complexes were built using psfgen package of VMD software^55^. Each of the trimolecular complex system was then immersed in a water box, and subsequently counter-ions were added in order to have a neutral system. Details about the simulation box size and the total number of atoms for each of the systems are presented in Supplementary Material. TIP3P^56^ parameters for water molecules and Charmm-27 force-field parameters for the proteins and peptides were used. Correct protonation state was assigned to all the protein and peptide residues using Propka software^57^. For TCR-unbound peptide-MHC complexes, we removed the TCR structure from the tri-molecular complex and performed MD simulations of only the peptide-MHC complexes. For simulations of peptide-MHC complex, we chose the chains corresponding to peptide and MHC molecules from the solved crystal structure.

We performed simulations both with/without TCR for the three systems (Table 1). Each system was energy minimized and slowly heated to 310 K in steps of 30 K with initial positional constraints of 50 kcal/(mol Å^2^) on carbon alpha atoms. Subsequently, positional constrains was slowly released in steps of 10 kcal/(mol Å^2^). Molecular dynamic simulation of 110 ns was performed in NPT ensemble with T = 310 K, and 1 atm pressure. Further simulation protocol details have been described in our previous works^58^,^59^. All-atom molecular dynamics (MD) simulations were performed employing NAMD^60^ software package on 64 processors cluster.

### Simulation analysis

MD trajectory of a total simulation time 110 ns, for each complex under investigation, was used for analysis. The stability of protein-peptide-protein complexes and peptide-protein complexes was evaluated by calculating the root mean square deviation (RMSD) values for the C-alpha atoms of residues during MD simulations (Supplementary Material). To estimate convergence of our MD simulations, we employed a novel Good-Turing statistical approach proposed recently by Koukos and Glykos^46^. This method allows estimating the probability distribution of unobserved configurations, p_unobserved_(RMSD), as a function of the RMSD distance between unobserved and observed molecular configurations in MD simulations (Supplementary Material). The hydrogen bonded (H-bond) interaction between peptide-protein or protein-protein residues pairs was calculated using a geometrical criterion, with a donor-acceptor cutoff distance of 3.1 Å and donor-hydrogen-acceptor cut-off angle 130 degree. H-bonds present for at least 20% of trajectory time length are reported. The aromatic stacking interaction between the residues pairs was calculated using EUCB software^61^ with following geometrical criteria: – maximum dihedral angle cut-off parameters between the planar/ring side chains of 30° – centroid distance cut-off between side chains 5.0 Å – persistence simulation time 20%.

The interaction energy between the two selected groups of atoms (for instance, between the peptide residues and HLA-DQ1 residues) was calculated by evaluating the non-bonded energy values comprising of Van der Waals and electrostatic energy, using the energy plugin of Namd software^60^. A cutoff distance of 12 Å was used for non-bonded interactions and for the electrostatic interaction we also adopted the particle mesh Ewald scheme^62^. The interaction energy scheme adopted in our calculations provides only a rough estimate in terms of enthalpic contributions to binding, as solvent effects are not included. Thus, interaction energy values obtained can be used only for the ranking the different systems based on their energy values.

Configurational entropy calculations using the quasi-harmonic approximation scheme^49^ were performed to investigate differences in protein flexibility and stability between the different protein-peptide complexes. From 110 ns MD simulation trajectory of each system, we extracted 5500 structures, and configurational entropy estimate was done by evaluating the covariance matrices of atomic fluctuations of selected residues, within a routine of CARMA software^63^.

Dihedral angles principal component analysis (dPCA) was performed on selected TCR and MHC class II binding site residues on MD simulation trajectory using CARMA software^63^. This resulted is a matrix containing the values of the top three principal components for each and every structure recorded in the trajectory. To perform cluster analysis, the method incorporated within the CARMA software uses a peak-picking algorithm that is applied to three-dimensional density distributions of the principal components derived from the MD trajectory. The classification of different clusters is done automatically using the density distribution threshold that can explain at least 80% of the original principal component map’s variance. Each classified clusters, represents a different number of structures (from the trajectory) that have values for their principal components corresponding to the specific point of the principal component plane. Cluster 1 corresponds to the most populated cluster with highest number of structures (from MD trajectory). A major limitation of this methodology it that it does not comprehensively assign each frame of a trajectory to a cluster. Indeed, the algorithm aims at efficiently identifying the most prominent molecular conformations.

## Additional Information

**How to cite this article**: Kumar, A. and Delogu, F. Dynamical footprint of cross-reactivity in a human autoimmune T-cell receptor. *Sci. Rep.* **7**, 42496; doi: 10.1038/srep42496 (2017).

**Publisher's note:** Springer Nature remains neutral with regard to jurisdictional claims in published maps and institutional affiliations.

## Supplementary Material

Supplementary Material

## Figures and Tables

**Table 1:** RMSD and buried surface area (BSA) calculations.

RMSD (backbone)	TCR			HLA-DQ1			Accessible surface area (Å^2^)
	Vα-chain	Vβ-chain	Total	α1-chain	β1-chain	Total	
UL15	1.3 Å	1.3 Å	1.6 Å	1.6 Å	1.4 Å	1.8 Å	1655 ± 63
PMM	1.1 Å	1.4 Å	1.9 Å	1.2 Å	1.4 Å	1.6 Å	1704 ± 64
MBP	REF	REF	REF	REF	REF	REF	1702 ± 63

RMSD values for Vα, Vβ chains of TCR (column 2, column 3) and α1, β1 chains (column 4, column 5) of HLA-DQ1. In column 6, we report accessible surface area values evaluated on MD simulation trajectory. The RMSD fluctuation values are reported with respect to MBP peptide complex as reference (REF).

**Table 2:** Hydrogen bonded interactions between TCR and HLA-DQ1.

TCR – MHC	UL15	PMM	MBP
Vα-DQ1 α1	**E98-R61** (3)	**E98-R61** (4)	**E98-R61** (2)
	—	—	E98-Q57 (1)
	—	—	K99-Q57 (1)
Vβ-DQ1 α1	**D55-K39** (2)	**D55-K39** (1)	**D55-K39** (2)
	E69-K75 (2)	Q48-H68 (1)	D54-K39 (3)
Vα-DQ1 β1	—	R51-E66 (4)	—
	—	R51-E69 (2)	—
Vβ -DQ1 β1	G26-Y60 (1)	—	—

Conserved interacting pairs are highlighted in bold, with the total number of interactions in ().

**Table 3:** Stacking interaction (pep-MHC) evaluation and configuration entropy calculations.

	Stacking interaction		Configurational Entropy (J/molK)	
	Peptide - DQ α	Peptide-DQ β	HLA-DQ1	TCR-MHC
UL15	F6 (CD1)-R61 (NE)	F10 (CD1):Y47 (CD1)	5672	8687
PMM	F6 (CD1)-R61 (NE)	R13 (NE)-P56 (CD)R13 (NE)-Y60 (CD1)	5965	8890
MBP	F6 (CD1)-R61 (NE)	H5 (CG)-R77 (NE)H5 (CG)-H81 (CG)P13 (CD)-Y60 (CD1)	5839	8877

**Table 4:** Peptide-MHC hydrogen bonded interactions and entropy evaluation in the presence and absence of TCR.

	UL15	PMM	MBP
a. Absent H-bond interactions in simulations without TCR			
DQ1 α-residues	αR61—D9 (P6)αC8—V7 (P4)αN11—D9 (P6)	αR61—D9 (P6)	αR61—N9 (P6)αH68—T12 (P9)αN62—F7 (P4)
DQ1 β-residues	βY37—Q12 (P9)βS74—R8 (P5)	—	βY9—N9 (P6)βH30—N9 (P6)
b. New H-bond interactions in simulations without TCR			
DQ1 α-residues	αY77—Q12 (P9)	—	αC8—F7 (P4)
DQ1 β-residues	βE66—R8 (P5)	βE66—R8 (P5)	βE66—K8 (P5)
c. Configurational entropy (J/molK)			
HLA-DQ1	6068 (7%↑)	6308 (6%↑)	6339 (8%↑)

(a) H-bond interactions absent in TCR-unbound simulations with respect to TCR–bound. (b) New interactions in unbound-TCR simulations. (c) MHC binding groove entropy analysis; an increase is reported in % with respect to TCR simulations.
