# Supplementary material for: Dynamical footprint of cross-reactivity in a human autoimmune T-cell receptor
Source: Sci Rep. 2017 Feb 14;7:42496. doi: 10.1038/srep42496 (PMC5307354; doi:10.1038/srep42496)
Supplement: Supplementary Material [file srep42496-s1.pdf]

Supplementary Material

Dynamical footprint of cross-reactivity in a human autoimmune T-cell receptor

Amit Kumar[1,2] and Francesco Delogu[1]

[1] Department of Mechanical, Chemical and Materials Engineering, University of Cagliari, via Marengo 2, 09123 Cagliari, Italy
[2] Biosciences Sector, center for advanced study research and development in Sardinia (CRS4), Loc. Piscina Manna, 09010 Pula, Italy

Table S1. Simulation of peptide-MHC complexes with and without Hy.1B11 TCR. We report total number of atoms and simulation box size in each of the system investigated.

Peptide – MHC complex	With Hy.1B11 TCR		Without Hy.1B11 TCR	
	Total atoms	Box-size	Total atoms	Box-size
MBP-HLA-DQ1	184164	[113 111 162]	48465	[79 67 107]
PMM-HLA-DQ1	184122	[113 111 162]	48450	[79 67 107]
UL15-HLA-DQ1	184151	[113 111 162]	48479	[79 67 107]

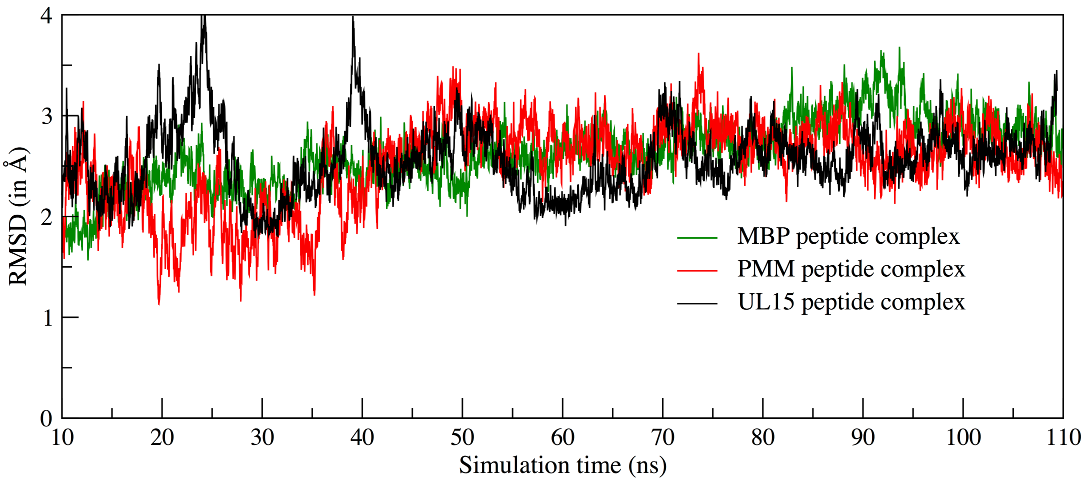

Figure S1. RMSD plot of C-alpha atoms for TCR-pep-MHC complexes.

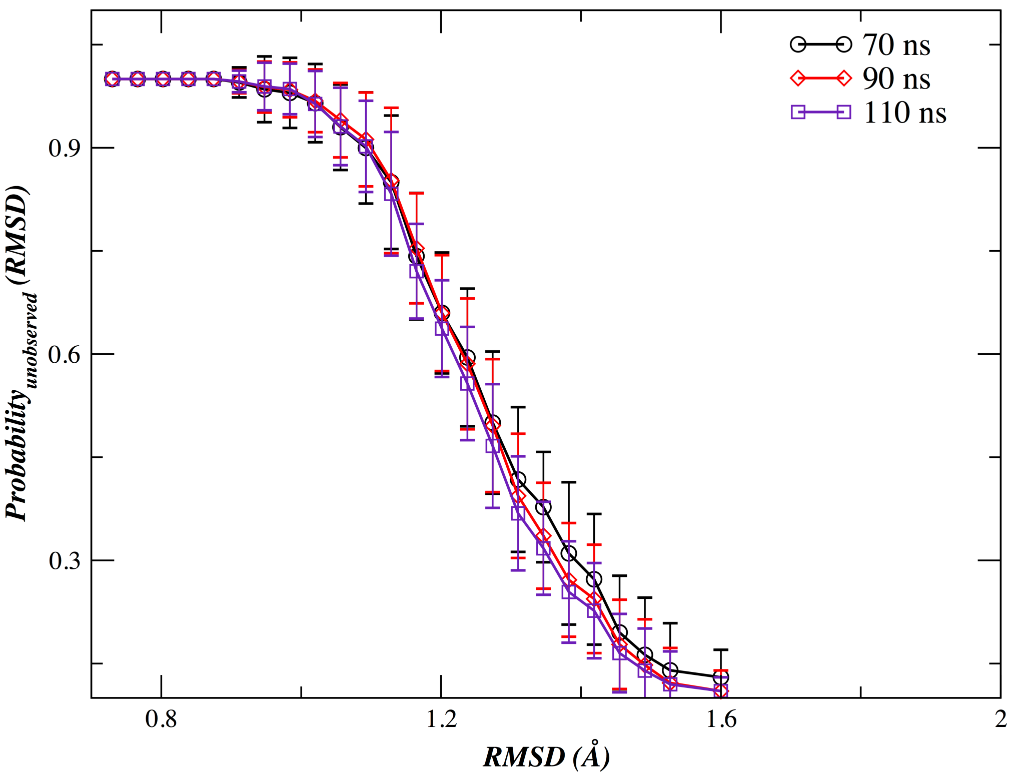

Figure S2. Good-Turing convergence test. Probability$_{\text{unobserved}}$ (RMSD) as a function of RMSD distance. For TCR bound MBP-MHC complex at different simulation time lengths.

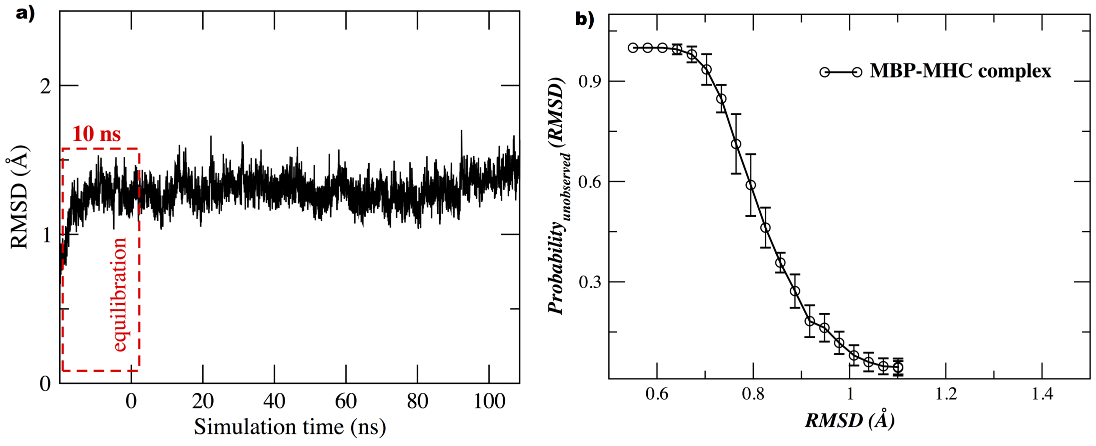

Figure S3. Stability and convergence plots. a) RMSD of C-alpha atoms of MHC binding groove residues for TCR-unbound simulations as function of simulation time. b) Good-Turing convergence test for MBP-MHC complex after 110 ns simulation time.

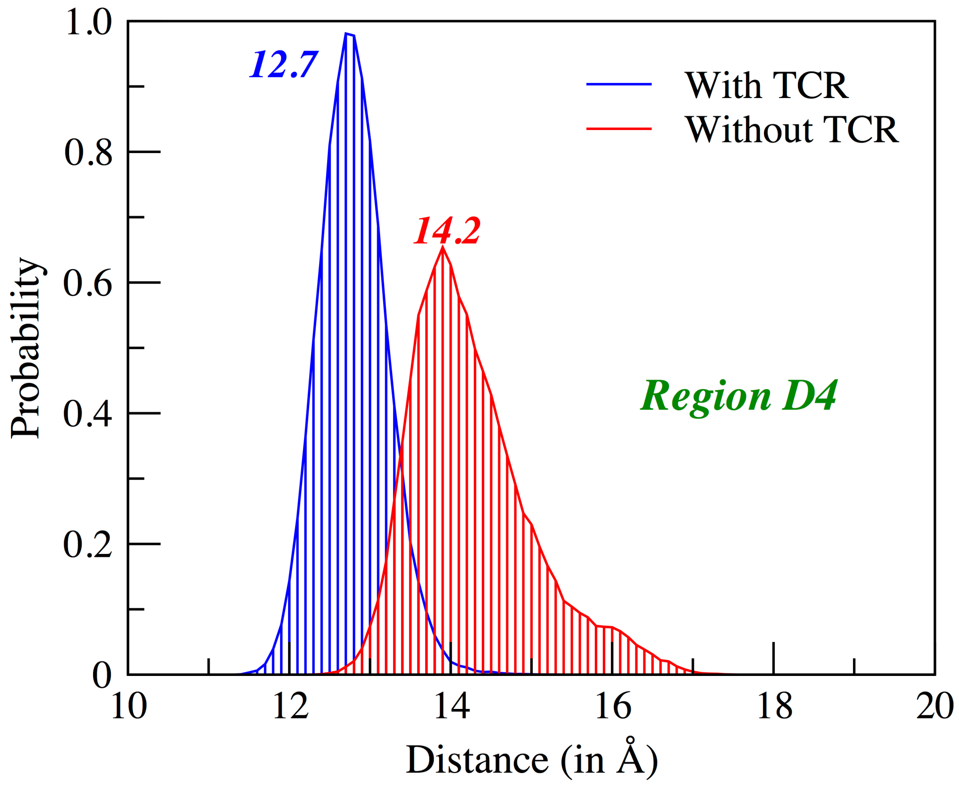

Figure S4. Region D4 distance probability plot for MBP bound MHC binding groove.

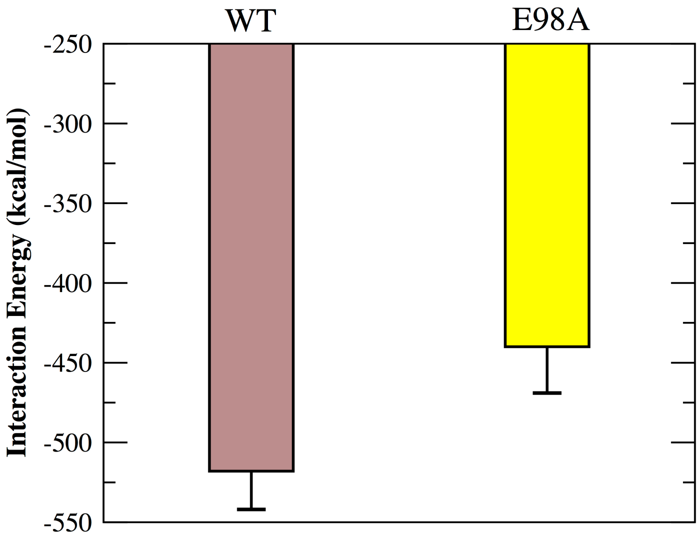

Figure S5. Interaction Energy plot. Comparison of interaction energy (Van der Waals and electrostatic energy) between TCR and MBP-MHC residues for WT and CDR3α E98A mutant TCR systems.
